# High Synteny and Sequence Identity between Genomes of *Nitrosococcus oceani* Strains Isolated from Different Oceanic Gyres Reveals Genome Economization and Autochthonous Clonal Evolution

**DOI:** 10.3390/microorganisms8050693

**Published:** 2020-05-08

**Authors:** Lin Wang, Chee Kent Lim, Martin G. Klotz

**Affiliations:** 1 Department of Biological Sciences, University of North Carolina, 9201 University City Boulevard, Charlotte, NC 28223, USA; linwangqd@gmail.com (L.W.); limcheekent@yahoo.com (C.K.L.); 2 School of Molecular Biosciences, College of Veterinary Medicine, Washington State University, 2710 Crimson Way, Richland, WA 99354, USA

**Keywords:** Ammonia-oxidizing bacteria, Evolutionary & genomic microbiology, Nitrification, Nitrogen cycle, *Nitrosococcus oceani*

## Abstract

The ammonia-oxidizing obligate aerobic chemolithoautotrophic gammaproteobacterium, *Nitrosococcus oceani*, is omnipresent in the world’s oceans and as such important to the global nitrogen cycle. We generated and compared high quality draft genome sequences of *N. oceani* strains isolated from the Northeast (AFC27) and Southeast (AFC132) Pacific Ocean and the coastal waters near Barbados at the interface between the Caribbean Sea and the North Atlantic Ocean (C-27) with the recently published Draft Genome Sequence of *N. oceani* Strain NS58 (West Pacific Ocean) and the complete genome sequence of *N. oceani* C-107, the type strain (ATCC 19707) isolated from the open North Atlantic, with the goal to identify indicators for the evolutionary origin of the species. The genomes of strains C–107, NS58, C-27, and AFC27 were highly conserved in content and synteny, and these four genomes contained one nearly sequence-identical plasmid. The genome of strain AFC132 revealed the presence of genetic inventory unknown from other marine ammonia-oxidizing bacteria such as genes encoding NiFe-hydrogenase and a non-ribosomal peptide synthetase (NRPS)-like siderophore biosynthesis module. Comparative genome analysis in context with the literature suggests that AFC132 represents a metabolically more diverse ancestral lineage to the other strains with C-107 and NS58 potentially being the youngest. The results suggest that the *N. oceani* species evolved by genome economization characterized by the loss of genes encoding catabolic diversity while acquiring a higher redundancy in inventory dedicated to nitrogen catabolism, both of which could have been facilitated by their rich complements of CRISPR/Cas and Restriction Modification systems.

## 1. Introduction

Strains of the bacterium *Nitrosococcus oceani* are Gram-negative marine gammaproteobacteria in the family *Chromatiaceae* [1]. The sequenced genome of *N. oceani* type strain C-107 provided a first snapshot of the genetic makeup of the species [2]. *Nitrosococcus. oceani* strain C-107 is omnipresent in the world’s oceans [3] and the type strain of the species in a genus of exclusively obligate aerobic and ammonia-dependent chemolithoautotrophs belonging to the functional guild of “ammonia-oxidizing bacteria” (AOB). This ammonia-catabolic lifestyle provides the energy and reductant required for the assimilation of carbon from carbon dioxide as the sole carbon source [4,5,6]. Oxidation of ammonia to nitrite (“nitritation”) occurs via a two-step pathway in the periplasm. Initially, ammonia is oxidized to the toxic intermediate hydroxylamine (NH_3_ + 2e^-^ + O_2_ + 2H^+^ → NH_2_OH + H_2_O) by ammonia monooxygenase (AMO), an integral membrane protein complex belonging to the family of Copper Membrane MonoOxygenases (CuMMO [7,8,9,10]) encoded by the *amoRCABD* operon [11]. Hydroxylamine is then converted to nitrite by the membrane-associated periplasmic hydroxylamine dehydrogenase (HAO), encoded by the *haoA* gene in the *haoA-haoB-cycA*-*cycB* operon [2,5,12]. While the 5*′*-end of the *haoB* transcript forms a stemloop that leads to two pools of steady-state *haoA* and *haoAB* mRNAs, which may be expressed into enzymes facilitating electron flow in opposite directions (hydroxylamine ↔ nitrite via an nitric oxide intermediate) [5,13], redox-active cytochrome *c* proteins encoded by the *cycA* and *cycB* genes have been implicated in the channeling of the four electrons extracted from hydroxylamine by HAO to the ubiquinone pool [14]; however, participation of cytochrome *c*554 (CycA) in this process has not been experimentally demonstrated [5,15]. Recent experiments implicate a hitherto unknown player aiding HAO in nitrite formation [16]. Two of the four electrons are recycled to AMO via a still unknown mechanism for the oxidation of ammonia, whereas the other two electrons are fueling the oxidative branch of the electron transport chain [4,5,15]. Nitrite is either released into the environment or subsequently reduced to nitrous oxide via nitric oxide by a pathway known as “nitrifier denitrification” [17,18,19] facilitated by a copper-dependent nitrite reductase (NirK) and a complement of enzymes that reduce nitric oxide, including nitric oxide reductases CytS (β-sheeted cytochrome *c*′-β, which is a member of high-spin cytochromes *c*) and the alternative heme-copper-containing nitric oxide reductase sNOR [2,4,5,15,20]. The existence of genes encoding the inventory required for complete oxidation of ammonia to nitrate in one organism, the “comammox” bacteria affiliated with the genus *Nitrospira*, has been demonstrated only recently [21,22,23,24]. The wide distribution of these complete nitrifiers has since been demonstrated for various habitats, although, so far, not identified in marine systems (e.g., [25,26,27,28]).

Because all known strains of *N. oceani* are distributed exclusively in marine environments albeit at an overall low abundance [1,3,29] and because the ocean is a major source of atmospheric nitrous oxide [30], a potent greenhouse gas [31], *Nitrosococcus* strains producing nitrogen oxides in environments with rising ammonia and urea levels contribute potentially significantly to the global climate crisis. This and the omnipresence of *N. oceani* in the world’s oceans generated the question whether different strains of the species isolated from different oceanic gyres differed in their genomic contents; in particular, genes encoding the catabolic inventory that facilitates transformations of nitrogen compounds. In this work, the genomes of three strains from different oceanic locations isolated into pure culture (C-27, AFC27, and AFC132) were sequenced and comparatively analyzed together with the sequences of the recently published genome sequence of *N. oceani* strain NS58 [32] and the previously analyzed type strain C-107 (ATCC 19707 = JMC 30415 = BCRC 17464 = NCIMB 11848 [2,33]).

## 2. Materials and Methods

### 2.1. Strain Isolation and Growth Conditions

**Figure 1:**
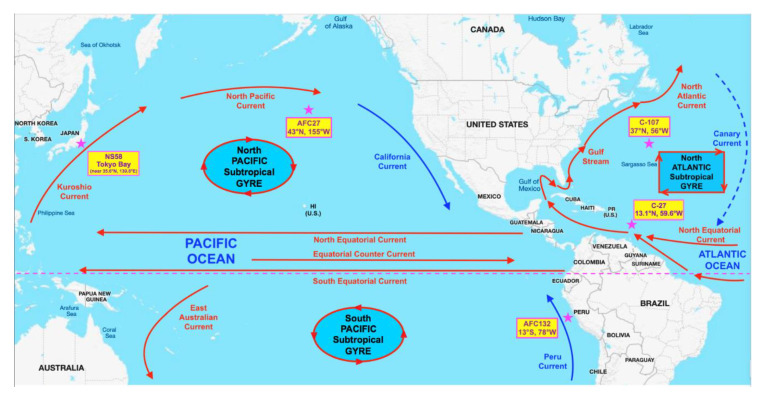
Locations of isolation of the studied *Nitrosococcus oceani* strains in the Pacific and Atlantic Oceans. Strains labeled with a “C-” were originally collected, enriched, and maintained by Stanley W. Watson (Woods Hole Oceanographic Institute, Woods Hole, MA, USA); strains labeled with a “AFC” were originally collected, enriched, and maintained by Angelo F. Carlucci (Scripps Institution, San Diego, CA, USA).

Strains used in this study were initially isolated as enrichment cultures from different oceanic gyres (Figure 1): C-107 from the open North Atlantic; C-27 from the coastal waters of the Southern North Atlantic; AFC132 from the South Pacific Gyre; and AFC27 from the East end of the North Pacific Gyre [3]. The enrichments were obtained under an agreement in the Nitrification Network collaborative from Bess B. Ward (Princeton University, Princeton, NJ, USA) and then isolated into pure culture in our lab using enrichment and growth conditions as described previously [1,2,34,35]. In brief, all *N. oceani* strains were maintained at 30 °C in the dark without shaking in liquid ammonium mineral salts media [34] amended with hydroxylamine at 100 µM final concentration to eliminate contamination [1]. The pH of cultures was adjusted daily to 8.0 using K_2_CO_3_ [2,34]. Cultures were propagated monthly by transferring 5-mL aliquots of culture into 100 mL of fresh media. Cells were checked under a light microscope at 1000× magnification routinely for uniform cell morphology [35]. As recently described by Dohra et al. [32], *N. oceani* strain NS58 was isolated into pure culture from Tokyo Bay sediment at the West East end of the North Pacific Gyre, which is clockwise connected to the location of AFC27 by the Kuroshio and North Pacific currents as well as the California and North Equatorial Currents (Figure 1).

### 2.2. DNA Extraction and Genome Sequencing

500 mL of mid-exponential phase culture were centrifuged (6000 × g, 15 min, room temperature) to harvest cells for genomic DNA extraction [1,35]. Resulting genomic DNA samples were quantified using a NanoDrop 2000 spectrophotometer (Thermo Fisher Scientific, Waltham, MA, USA) and checked for integrity by (1%) agarose gel electrophoresis. About 1 µg of high quality gDNA was obtained from each culture for genome sequencing.

Draft genomes of strains C-27 and AFC132 were generated at the Center for Genome Research and Biocomputing (CGRB) at Oregon State University using Illumina MiSeq Sequencing platform using TruSeq techniques for library construction with size selection at the default value of 300–400 bp, which produced 27,907,041 paired ends reads for strain C-27 and 28,295,664 reads for strain AFC132 after quality check and trimming. The whole genome of AFC27 was shotgun sequenced at the J. Craig Venter Institute (http://www.jcvi.org; Rockville, MD, USA) using 454 FLX Titanium technology funded by Marine Microbial Genome Sequencing Project of The Gordon & Betty Moore Foundation (http://www.moore.org; Palo Alto, CA, USA). After quality trimming of the draft sequence, 30,135 reads were generated and assembled into 49 contigs and 12 scaffolds with no gaps. The total assembly gap length was 8252 bp with an estimated total genome sequence length of 3,480,059 bp [36].

### 2.3. Genome Assembly, Annotation, and Bioinformatics Analyses

After quality checks, adaptor trimmed raw reads from C-27, AFC27, and AFC132 genome templates were used for de novo assemblies performed with the CLC Genomics Workbench v.12 (CLC Bio/Qiagen, Hilden, Germany), the NCBI Prokaryotic Genome Annotation Pipeline (PGAP) [37], and the Mauve alignment package (Mauve v. 2.4 [38,39,40], whereas the RAST SEED server (v. 4.0 [41]), RASTtk [42] and OrthoVenn2 [43] were utilized for comparative annotation. To facilitate our comparative study, the IslandViewer4 software package was utilized to predict and analyze genomic islands [44] and antiSmash 2.0 [45] was employed to analyze secondary metabolite biosynthesis gene clusters. The web server PHAST (PHAge Search Tool) [46] was used to search for prophage sequences inside the bacterial genomes. Genetic relatedness among *N. oceani* species was comparatively assessed by pairwise calculating the average nucleotide identity (ANI) between the analyzed genomes ([47] http://enve-omics.ce.gatech.edu/ani/). Sequence Data Analysis Bioinformatics Software by Geneious (R10; Biomatters, San Diego, CA, USA) was utilized for further analysis of the genomes. The sequence synteny web server SyntTax was used to assess the orthology of genomic regions and to predict functional relationships between genes. CRISPR/Cas systems were identified in the *N. oceani* genomes by employing the web tool CRISPRFinder [48]. For identification of Toxin–antitoxin systems, RASTA-Bacteria [49] and TADB were utilized [50]. Phylogenetic relationships among the 5 investigated strains of *N. oceani* were determined based on one of their two identical 16S rRNA gene sequences in the context of 16S rRNA gene sequences from selected bacteria in the order Chromatiales obtained from NCBI Genbank. Nucleic acid sequence alignments, generated using MUSCLE (https://www.ebi.ac.uk/Tools/msa/muscle), were subjected to Bayesian inference methods using BEAST v. 1.8 [51]. Bayesian analysis employed the following modeling strategy: HKY substitution, a Gamma + Invariant sites heterogeneity model with 4 gamma categories and a strict clock model. The inferred tree topology was assessed with 10,000,000 iterations for Bayesian phylogenies minus a burn-in of 20% of the total.

### 2.4. Genome Sequence Deposits

The Whole Genome Projects for *N. oceani* strains C-27, AFC27, and AFC132 have been deposited in the NCBI GenBank database with the accession numbers NZ_JPGN00000000, NZ_ABSG00000000, and NZ_JPFN00000000, respectively. BioSample, BioProject and Assembly accession are available through the NCBI web portal [52].

## 3. Results and Discussion

### 3.1. General Genome Characteristics of Nitrosococcus oceani Strains

The *Nitrosoccus oceani* strains compared in this study were isolated from different gyres of the Atlantic and Pacific Oceans [32,53] (Figure 1). Strains of widely distributed marine bacterial species such as *Prochlorococcus marinus* or *Photobacterium profundum* but not *Crocosphaera watsonii* were found to exhibit moderate to high cross-species genomic diversity [54,55,56]. *Nitrosococcus oceani* has also been reported in the literature as being omnipresent in the world’s oceans, initially characterized by noticeable differences between the sequences of their 16S rRNA genes [3]. In contrast to Ward and O’Mullan [3] working with enrichment isolates sampled from diverse oceanic gyres, we succeeded in enriching several of these *N. oceani* isolates into pure cultures and the alignment and analysis of determined 16S rRNA gene sequences of strains C-107, C-27, AFC27, and AFC132 in pure culture yielded near identical sequences. This and because we are generally interested in the evolution of molecular diversity in ammonia-oxidizing bacteria including *N. oceani* [1], we decided to determine the entire genome sequences and set out to examine the extent of genomic diversity within the *N. oceani* lineage by focusing on these four strains originally isolated from the Atlantic (C-107 and C-27) and the Pacific (AFC27 and AFC132) Oceans. While working on this project, Dohra et al. [32] identified and published the draft sequence of *N. oceani* strain NS58 using as reference template the sequences of the four *N. oceani* genomes we had deposited in Genbank to make the data publicly available. To strengthen the conclusions based on our genome analyses, we decided to realign the sequence reads of the NS58 genome publicly deposited by Dohra et al. after publication [32] and included the alignment constructed with slightly different methods in our analysis. The strains’ chromosomal genome sizes are similar to each other with sizes of about 3.5 Mb (Table 1). The GC content of all five genomes is approximately 50%; however, there is a trend of increasing GC content (AFC132 → C-27 → AFC27 → C-107/NS58) that correlates with the number of encoded CDS: indirectly through decreasing numbers for total CDS and singletons and directly through an increasing number of orthologous clusters (Table 1).

Surprisingly, in light of the disconnected points of origin, the alignment of the genomes from different strains revealed a high degree of synteny with only a few major rearrangements in the genomes of AFC27.

**Figure 2:**
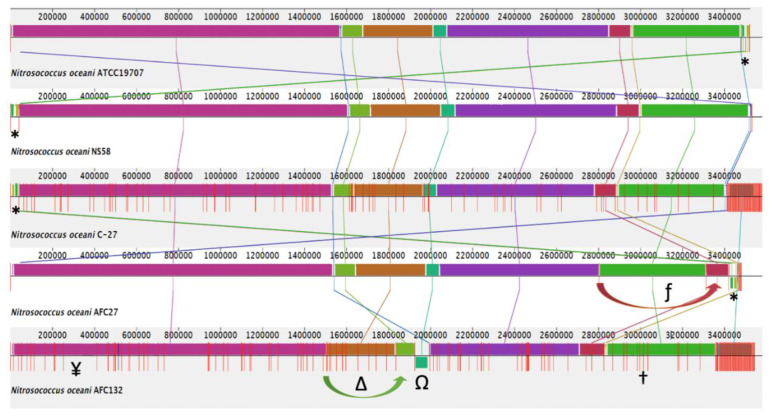
Alignment of the genome sequences from five *Nitrosococcus oceani* strains C-107, NS58, C-27, AFC27, and AFC132, using Mauve 2.4 [37], the progressiveMauve aligner [40], and the Mauve Contig Mover module [39]. *, Plasmid A sequence; Ω, inverted AFC132 contig_72; ∆, translocated AFC132 contig_71; ƒ, translocated AFC27 contig_NS_D5995302.1; †, NiFe-hydrogenase metallocenter assembly protein HypFCDE on AFC132 contig_122 (3,080,491–3,085,122); ¥, MbtH family NRPS protein HW44_RS01565 (344,153–344,419).

In the AFC132 genome, an inversion in contig_72 with 61,257 bp in length and a translocation of contig_71 with a length of 93,149 bp were observed. A closer look at the gene content of both contigs revealed that the region of the genome was rich in transposase-encoding genes: contig_71 contained several genes encoding for transposases of the IS5, IS630, and other families; several genes on contig_72 encoded transposases of the IS200/IS605 family as well as several restriction endonucleases, in support of the modeled rearrangements shown in Figure 2. In the AFC27 genome, a translocation of contig_NS_D5995302.1 with a length of 106,475 bp was observed. In addition, the original reads deposited for the NS58 genome project (GCA_007990675.1) were realigned using the De Novo Sequencing tool of CLC Genomics Workbench v. 12 (CLC Bio/Qiagen, Hilden, German) and the resulting contigs were mapped to the reference genome of C-107 using CLC Genome Finishing module. Where needed, gaps between contigs were filled with “N”s and the resulting assembly was then used for multiple genome alignment analysis (Figure 2) The identified translocations of large segments in the AFC27 and AFC132 genomes were confirmed by overlapping reads across neighboring segments (Figure 2).

**Figure 3:**
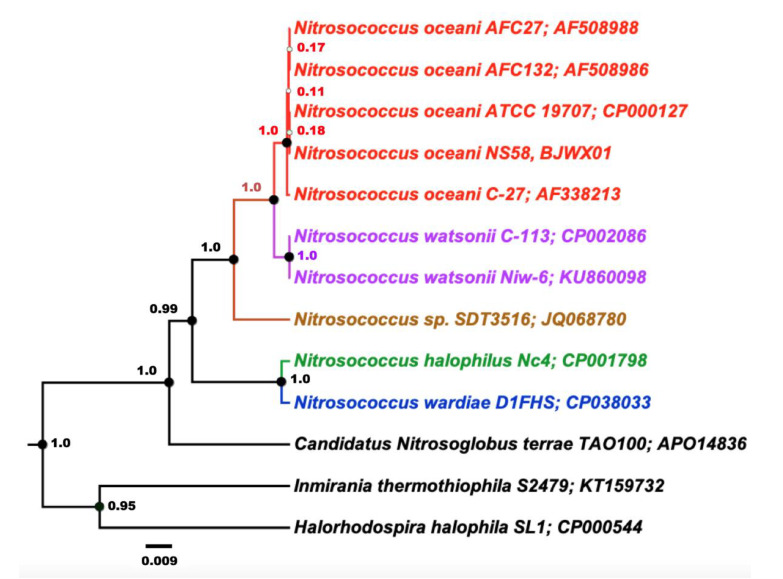
Inferred phylogenetic relationships among the five investigated strains of *Nitrosococcus oceani* based on one of their two identical 16S rRNA gene sequences in the context of Chromatiales bacteria. The phylogenetic tree was constructed based on an alignment of 16S rRNA gene sequences subjected to Bayesian inference employing a Gamma + Invariant sites heterogeneity model with four gamma categories with a strict clock model. Node values are Bayesian posterior probabilities based on 10,000,000 iterations, minus a burn-in of 20% of total. The scale bar represents 0.009 changes per nucleotide position. Colors indicate established taxonomic groups.

Analysis of an alignment of 16S rRNA gene sequences using Bayesian inference demonstrated the expected close relationship between the investigated strains of *N. oceani* when compared with pertinent 16S rRNA gene sequences in genomes of selected Chromatiales bacteria including the type strains of other *Nitrosococcus* species (Figure 3).

This confirmed that the genomes of strains C-107, NS58, C-27, AFC27, and AFC132 contained near-identical 16S rRNA sequences, two identical sequences in each genome, which is in agreement with a previous study [1].

We calculated almost identical ANI values close to 100% for the pairwise comparison of the genomes of C-107, NS58, C-27, and AFC27, and the ANI for the pairwise comparison of the AFC132 genome with each of the four other genomes was greater than 98.2% (Table 2). Such low level of sequence diversity at the strain level was also reported for strains within other marine bacterial species including *C. watsonii* [54,57], *Vibrio cyclitrophicus* [58], or *Alteromonas macleodii* [59]. For example, strains of *A. macleodii* exhibited high genome sequence conservation as demonstrated by two-way ANI values near 100% (i.e., 99.98%) for any two *A. macleodii* isolates (i.e., the strains U4 and U7 from the same clonal frame); however, these were all isolated from the Mediterranean Sea [59]. In contrast, *N. oceani* strains C-107, NS58, C-27, AFC27, and AFC132 were isolated from different locations in the Atlantic and Pacific Oceans that have been physically separated during evolutionary times by the landmass of the Americas and their calculated two-way ANI values were also near 100% (Table 2), which suggests direct clonal history.

**Figure 4:**
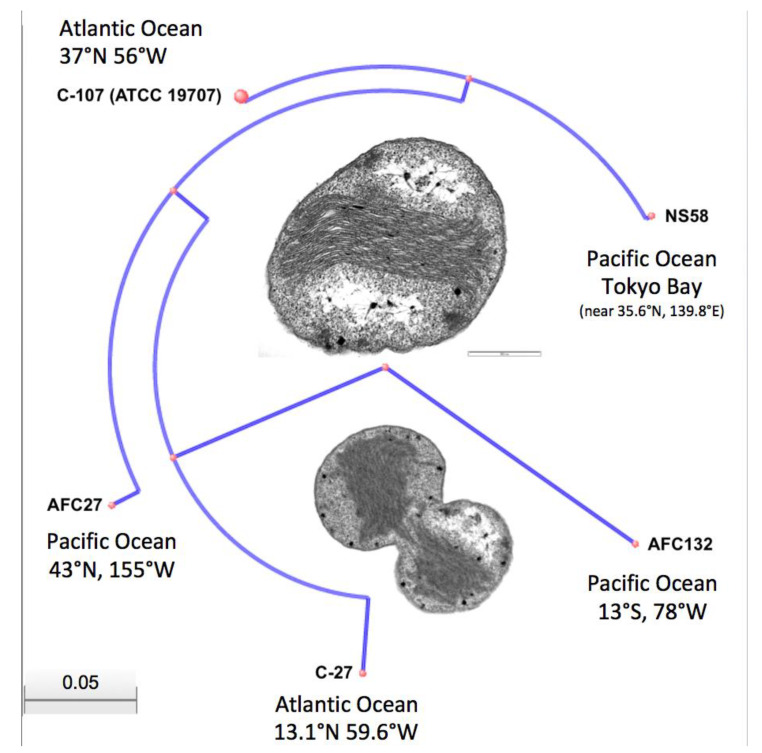
Dendrogram based on NCBI genomic BLAST analysis. Inserts show Transmission Electron Micrographs (TEM) of resting and dividing cells of *Nitrosococcus oceani* type strain C-107. TEMs were generated in the Electron Microscopy facility, University of Wisconsin-Madison, as described previously [2].

While the high-quality draft sequence assemblies of the AFC27, NS58, C-27, and AFC132 genomes are based on a varying number of scaffolds (ranging from 12 to 630; see also Figure 2) and only the C-107 genome has been closed and finished, their comparison using ANI analysis is warranted [47,60]. The ANI values obtained for the pairwise comparisons (Table 2) are well above the cut-off of 95% for delineating species [47], thereby confirming the assignment of all five strains to the same species, *N. oceani*, and outside of the next closest related species, *Nitrosococcus watsonii,* with an ANI value of 89.3% compared to the genome of *N. oceani* C-107 [1,35], which confirmed the analysis of 16S rRNA gene sequences (Figure 3). Employment of the NCBI genomic BLAST tool for comparison of the deposited genome sequences of the five *N. oceani* strains (https://www.ncbi.nlm.nih.gov/genome/?term=Nitrosococcus+oceani) resulted in a dendrogram that visually captured the results of the ANI analysis described above (Figure 4).

### 3.2. Molecular Diversity Among the Nitrosococcus oceani Genomes

**Figure 5:**
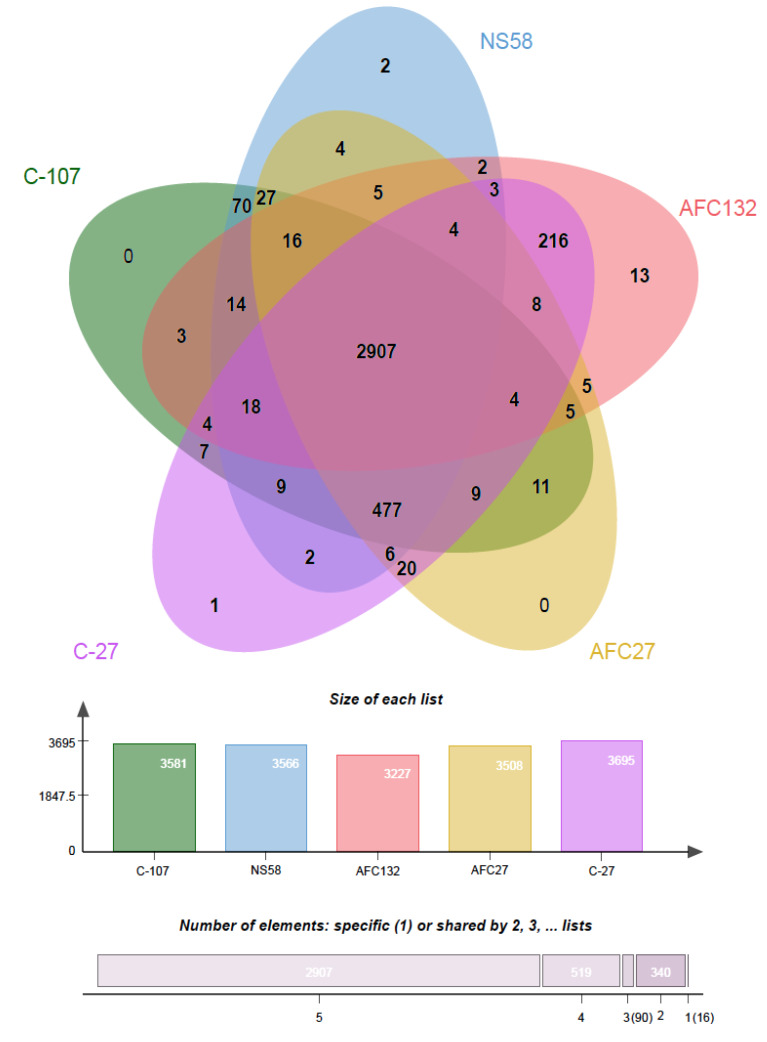
Venn diagram drawn using OrthoVenn2 [43] showing the genome comparison of five *N. oceani* strains. Pairwise sequence similarities between all input protein sequences were calculated with an e-value cut-off of 1e−5. An inflation value (*I*) of 1.5 was used to define orthologous cluster structure. The core genome of the *N. oceani* species consists of 2907 CDS. The species forms 3872 CDS clusters, 980 orthologous clusters (containing ≥2 species), and 2892 single-copy gene clusters.

An analysis of orthologous gene clusters (OrthoVenn2 [43]) revealed that a core of 2907 orthologous genes are shared among the genomes of the five *N. oceani* strains (Figure 5). In addition, the number of orthologous gene clusters present in the genomes of C-107, NS58, C-27, and AFC27 but absent from the genome of AFC132 is 477 (Figure 5).

In addition, the genomes of strains C-107 and NS58 share the highest number of orthologous gene clusters (70) not present in the other genomes, followed by the sharing of orthologous gene clusters by C-107, NS58, and AFC27. No unique orthologous gene clusters were detected in C-107 and AFC27 whereas the genomes of strains C-27, NS58, and AFC132 contained 1, 2, and 13 unique genes, respectively (Figure 5). This result supports the general analysis of gene content (genome BLAST) as well as the alignments of the genome sequences from strains C-107, NS58, C-27, and AFC27, which demonstrated a high level of conservation in sequence identity between these strains, including the arrangement of genes in the respective genomes (Table 2 and Figure 2). As predicted by IslandPath-DIMOB and SIGI-HMM modules of IslandViewer 4, the C-107 and NS58 genomes differ from the other three genomes by the presence of a large genomic island encompassing a 24-kb DNA region (738646–761019) that contains 26 genes (see below). Another genetic difference between the strains was detected in the region that aligns with the NOC_RS12615 and NOC_RS12620 genes in C-107, which both encode Fis transcriptional regulators with moderate sequence identity to each other. The NOC_RS12615 and NOC_RS12620 genes in C-107 are combined to form a fused single gene (HW44_RS11470) in the AFC132 genome, which is predicted to encode a sigma-54 type transcriptional regulator in the same class of these regulators found in the C-107 genome. Interestingly, the C-27 and AFC27 genomes lack the DNA segment between the NOC_RS12615 and NOC_RS12655 homologs present in C-107 resulting in the absence of five genes and the fusion of the NOC_RS12615 and NOC_RS12655 homologs to form a single gene predicted to encode a flagellin protein in the two strains. This leaves the genomes of C-27 and AFC27 being closer related to one another than either is related to AFC132 (Figure 4 and Figure 5). A key feature distinguishing the AFC27 and C-27 genomes from one another is a gene encoding a DNA polymerase III beta subunit domain that is inserted within NOC_RS02200, which is predicted to encode a DUF1722 domain-containing protein. Although the AFC132 genome retained a high level of genomic synteny in comparison with the other genomes, our analysis suggest that the occurrence of the reported genome rearrangement (Figure 2) affected the magnesium/cobalt transporter *corA* homologous gene NOC_RS07660 in AFC132 leading to the deletion of the 5′-end of the gene, thereby causing deletion of the N-terminus of the CorA protein that is expressed in the other *N. oceani* strains.

#### 3.2.1. Extra-Chromosomal DNA (Plasmid)

The genomes of strains C-27, NS58, and AFC27 appear to include a plasmid that is identical or near identical in sequence to plasmid A in the C-107 strain. In detail, the plasmid of C-27 corresponded to plasmid A of C-107 with 100% nucleic acid identity, except for a deletion of 420 nucleotides (corresponding to C-107 plasmid A sequence positions 17241–17660). Interestingly, the 40,421-bp-sized plasmid in AFC27 was sequence-identical to plasmid A in C-107 and C-27, albeit with one additional nucleotide. Alignment of the sequences of C-107 plasmid A with plasmid A identified by Doha et al. [32] in strain NS58 confirmed their near identity. In contrast, not a single read of sequencing data for the AFC132 genome could be mapped onto the C-107 plasmid A template sequence. These results suggest that plasmid A is present as an independent replicon in all strains but AFC132, which resembles the situation of *A. macleodii* whose isolates presumed to be derived from a close common ancestor exhibit low genome sequence diversity but do not all carry plasmids [56].

#### 3.2.2. Restriction Modification Systems

Restriction endonucleases (RE) are employed by bacteria to defend themselves against invasive foreign DNA by recognizing particular sequence patterns of DNA and /or its methylation state and cutting the DNA into shorter segments. Host DNA with sequence patterns recognized by REs can be protected by methylation, thus making them impervious to the actions of the REs thereby enabling REs to differentiate between host and foreign DNA. There are four types of restriction systems (RM), which are designated I–IV [61].

According to the REBASE database [62], the chromosome of C-107 carries 20 methyltransferase gene clusters with another one on its plasmid. Analysis of the C-27, NS58, and AFC27 genomes revealed that all three strains contain the same RM inventory as on the chromosome of C-107. In contrast, the total number and types of RM systems encoded in the AFC132 genome is smaller and some systems are incomplete. For instance, homologs of a Type I RM system encoded by NOC_RS06315-RS06360 in C-107 are also encoded by the genomes of strains C-27, NS58, and AFC27 but completely absent from the genome of AFC132. Other Type I RM systems encoded by the genomes of strains C-107, NS58, C-27, and AFC27 were conserved in the AFC132 genome for homologs of modification (M) subunit-encoding genes (i.e., NOC_RS02455 and NOC-RS15365) but the homologs of the pertinent specificity (S) subunit-encoding genes were absent from the AFC132 genome. In another configuration, the homologs of restriction (R) (NOC_RS15500) and S (NOC_RS15505) subunit-encoding genes of another RM system were present in all five genomes, although interrupted in the AFC132 genome by a gene (HW44_RS14445) encoding a protein with a “phage abortive infection” (Abi) system domain. Abi systems are involved in defense responses against phage attack and they operate similar to toxin–antitoxin systems, by which the toxin is neutralized by a cognate antitoxin [63,64]. In AFC132, the gene upstream of the Abi-like encoding gene is annotated as a gene encoding a hypothetical protein, which might code for the antitoxin part of the system. Notably, the AFC132 genome also contained unique genes implicated in DNA modification such as HW44_RS13205, encoding the S subunit of a Type I RM system and HW44_RS09935, encoding a restriction endonuclease. Interestingly, a gene cluster encoding a Type I RM system in the genomes of C-107, NS58, C-27, and AFC27 (NOC_RS06540-RS06570 homolog) is replaced in the genome of AFC132 by a gene cluster encoding a Type III RM system. This replacement was likely the outcome of a horizontal transfer event as these RM systems mobilize independent of accessory mobile elements [65]. Another Type III RM system in C-107 (NOC_RS06315-RS06335) conserved in the genomes of strains C-107, NS58, C-27, and AFC27 was represented in AFC132 albeit only by a truncated homolog of NOC_RS06330. Other gene clusters, encoding Type II and Type III RM systems in all five genomes, were found to be conserved (content and synteny) in the genomes of strains C-107, NS58, C-27, and AFC27 but translocated to different positions in the AFC132 genome.

#### 3.2.3. CRISPR/Cas Systems

The CRISPR/Cas system acts as a defense mechanism in archaea and bacteria functionally analogous to immunity systems, which give the organisms protection against invasion of previously encountered foreign genetic elements such as phages and plasmids [66,67]. This system is widespread among archaea (about 90%) and bacteria (about 40%) [68]. CRISPR loci consist of arrays of direct repeats separated by spacer DNA and are usually located close to CRISPR-associated (*cas*) genes that code for heterogeneous families of proteins [69]. The length of the direct repeats can vary between 23 and 47 bp while the length of the spacer varies between 21 and 72 bp [68]. The CRISPR loci are formed by the incorporation of new spacer DNA derived from the invading foreign DNA elements into the CRISPR thereby conferring adaptive immunity [64]. Exposure to different foreign invading DNA agents can hence result in the build-up of CRISPR loci that provide immunity even between closely related microbial strains [68,70,71].

In general, the genomes of the *N. oceani* strains C-107, NS58, C-27, and AFC27 harbor similar CRISPR/Cas systems (Table 3). Although AFC132 contains CRISPR/Cas system of the same Subtype I-F [72] and exhibits the same direct repeat length (28 bp), it has a slightly different direct repeat consensus sequence, a much longer CRISPR length (2248 bp versus 387 bp) and about a six-fold higher number of spacers (Table 3). In particular, the genetic organization of CRISPR/Cas system in the AFC132 genome compared with the other genomes is different. While the Cas gene cluster in AFC132 is organized in a unidirectional order, the Cas gene clusters in the other genomes are divided into two parts in a bidirectional manner with the *cas* genes and *cys* genes being juxtaposed. The alignment of all five genomes indicated that the shared CRISPR/Cas clusters are located at the same positions in the genome; suggesting that the diverging CRISPR/Cas cluster is the outcome of a replacement by heterologous recombination, presumably before genome diversification ensued and the established omnipresence of *N. oceani* represented by the present array of strains was established. The significant reduction in CRISPR cluster length in C-107, NS58, C-27, and AFC27 compared to AFC-132 could be the result of functional pressure exercised by extensive editing and concomitant loss to a point where further loss would make them susceptible to viral attack.

#### 3.2.4. Toxin–Antitoxin Systems

The stable retention of plasmids often correlates with the function of toxin–antitoxin systems because the antitoxin function is usually encoded on the plasmid; however, our analysis identified gene clusters encoding toxin–antitoxin (TA) systems in the chromosome of all *N. oceani* strains. The roles of toxin–antitoxin systems encoded in the chromosome are not well understood; a few reports suggest that toxin–antitoxin systems encoded in chromosomes can play other biological roles such as increasing the tolerance to antibiotics [73], protecting against phage attack [74] or participating in post-transcriptional regulation [75].

Interestingly, our finding that C-107, NS58, C-27, and AFC27 genomes differ from the AFC132 genome in their content of encoded toxin–antitoxin systems appears to correlate with plasmid residence. Generally, the genomes of strains AFC132 and C-27 encode fewer TA systems and the AFC132 genome contains either incomplete clusters or genes separated or translocated compared to the conserved location in the other genomes. Genes encoding protein families belonging to Type II toxin–antitoxin systems and addiction modules are highly abundant and widespread in genomes of all five strains. For example, the genomes encode toxins and antitoxins associated with the HicA/B, PemK/MazF, Phd/YefM, RelE/ParE, and VapB/VapC protein families; addiction module toxins in the HicA and Txe/YoeB families; toxins of the HipA, HigB, PIN, and RatA protein families; antitoxins of the Type II toxin–antitoxin system prevent-host-death family; and a mRNA interferase toxin in the RelE/StbE family. Toxins and antitoxins in the BrnT/A protein family were only found in C-107, NS58, and AFC132. Additionally, a gene predicted to encode a nucleotidyl transferase in the AbiEii/AbiGii toxin family of Type IV toxin–antitoxin systems was identified on the plasmids of strains C-107 (NOC_RS00145), NS58 (NONS58_RS00145), C-27 (IB75_RS17280), and AFC27 (NOC27_RS15820). Furthermore, homologs of the contiguous genes NOC_RS11465 and NOC_RS11470, encoding a PhoX family phosphatase CDS), present in all five genomes, are interrupted in the AFC132 genome by a cluster of three genes, HW44_RS10340-RS10350, predicted to encode a hypothetical protein, a Type II toxin–antitoxin system in the Phd/YefM antitoxin family and a putative toxin–antitoxin system of the PIN family. Furthermore, the C-107 genome contains three Type II (Vap-family) toxin–antitoxin system-encoding genes at NOC_RS00530, NOC_RS02910, and NOC_RS13540, which are conserved in all of the other genomes at corresponding locations except for the missing homolog of NOC_RS00530 in the genome of AFC132.

#### 3.2.5. Mobile Genetic Elements and Genomic Islands

Initially, the publication of the genome of *N. oceani* C-107 genome revealed a high number of mobile elements, especially transposases [2]. In this study, the examination of other *N. oceani* strains confirmed the residence of numerous mobile elements in their genomes. Although we could not assess the full complements of the mobile elements in all strains due to the difference in finishing status of the genome sequences and the repeat nature of many of the mobile elements, we were able to examine the mobile elements identified, including unique insertions into genomes of the different strains.

Although the high-quality draft status of the NS58, C-27, AFC27, and AFC132 genomes precludes exclusive statements about the absence of transposon elements present in C-107, we nevertheless made some interesting observation regarding genes that are present in the genomes. Genomic comparison revealed that the majority of the transposases of IS3, IS5, IS200/IS605, and IS630 families identified in the C-107 genome were found in the other strains as well. Analysis of genome alignments also revealed that the distribution of transposase genes was most similar among C-107, NS58, C-27, and AFC27 genomes while many of their locations in the AFC132 genome were different. Nevertheless, the transposases encoded in the AFC132 genome belonged to the same families found in the genomes of the other strains. Some genes encoding transposases of the same family (e.g., IS605 OrfB) in all *N. oceani* genomes were distributed differently suggesting random insertion or jumping events. Several copies of genes predicted to encode IS200/IS605 family element transposase accessory protein TnpA in the genome of strain C-107 (WP_002810279.1) are present in the NS58, AFC27, and AFC132 genomes but absent from the C-27 genome. However, the genome of C-27 contains genes predicted to encode transposases from the S21 transposase family (IB75_RS16105 and IB75_RS16370), the IS256 family (IB75_RS18435), and the IS481 family (IB75_RS15775 and IB75_RS15965), which are absent from the genomes of C-107, NS58, AFC27, and AFC132. Furthermore, genes encoding transposases in the IS4, IS1595 and IS5/IS1182 families were only observed in the genomes of strains AFC132 and C-27. In addition to single transposase insertions in the genomes, we observed large aggregations of mobile elements within small regions. Examples of these are homologous regions of NOC_RS00445 (integrase) to NOC_RS00485 and NOC_RS17160 to NOC_RS00590 (IS630 family transposase CDS), which are conserved in the genomes of C-107, NS58, C-27, and AFC27 but not in AFC132. These conserved insertions constitute either horizontally acquired genomic islands in the ancestor of the C-107, NS58, C-27, and AFC27 genomes after delineating from AFC132 or the islands were lost from the AFC132 genome after its delineation from the common ancestor genome. The detected NOC_RS00445-RS00485 gene cluster includes several hypothetical protein-encoding genes as well as a gene encoding a TOPRIM domain-containing protein.

A unique cluster of insertion elements is observed in the AFC132 genome at a position between gene homologs of NOC_RS15510 and NOC_RS15515 in the other four genomes. This unique region contains a few putative coding genes including one that encoding a DEAD/DEAH box helicase-like protein and another encoding an excisionase family DNA-binding domain. This region is marked as a genomic island predicted by using IslandViewer4 [44] Additionally, genes predicted to encode transposase in the IS1 family (HW44_RS17215, HW44_RS17265, and HW44_RS17705), IS110 family (HW44_RS16715, HW44_RS16590, HW44_RS18070, and HW44_RS18130), ISAzo13 family (HW44_RS15500, HW44_RS15595, and HW44_RS18005) and ISNCY family (HW44_RS15525 and RS1562) are uniquely present in the genome of AFC132 but not the other *N. oceani* strains.

The genome of AFC132 includes one intact prophage gene cluster as identified by the PHAST web server [46]. In contrast, only one remnant prophage gene cluster NOC_RS09985-RS09995 was discovered in the closed C-107 genome [2], which we have identified in the same location in the C-27, NS58, and AFC27 genomes but not in AFC132. Interestingly, a DNA region containing phage integrase (HW44_RS16220) is only present in AFC132.

Bioinformatics analyses revealed putative genomic islands in the genomes of C-107, NS58 and AFC27. The most conspicuous genomic island found only in the C-107 and NS58 genomes encompass a 24-kb DNA region (738,646–761,019) at identical locations in the C-107 and NS58 genomes that represents a genomic island with 26 genes encoding mostly hypothetical proteins but also putative DNA processing proteins providing mobile element functions such as invertase, transposase, phage integrase, and resolvase-like protein. The island also encodes a copy of the molecular chaperone DnaK, exonucleases, and a pair of genes encoding a two-component regulatory system. The genes flanking this genomic island in the C-107 and NS58 genomes are conserved in the other three *N. oceani* genomes where they sandwich a mobile element thereby identifying this location as an insertion before strain delineation.

The bioinformatics analyses also predicted a 62-kb DNA genomic island in AFC27 (1,180,844–1,242,823) that contains 59 genes (including a gene region encoding transposases and an integrase), of which a 21-kb segment (1,199,513–1,220,518) overlapped at its location with another genomic island in the C-107 (but not the NS58) genome (NOC_RS05945-RS06080) predicted to encode 28 genes including a Txe/YoeB toxin addiction module, transposase, and genes encoding NADH-ubiquinone oxidoreductase subunits. These could be “flexible genomic islands”, which are defined as genomic regions that have homologous position in the genomes, have similar inferred function but contain different sets of genes [76].

### 3.3. Comparison of the Metabolic Capacity Between the Nitrosococcus oceani Genomes

#### 3.3.1. Nitrogen Metabolism Inventory

Given the importance of *N. oceani* in the nitrogen cycle, an in-depth comparative analysis of nitrogen metabolism-related genes was performed in this study. Generally, gene clusters involved in the nitritation and denitrification pathways such as *amoCAB* and *haoABcycAB* as well as *nirK* and *norCB* [5,77] are conserved in all of the *N. oceani* strains. The genomes also encode nitric oxide reductases CytS and sNOR [15]. The role of the CytS is likely to mitigate toxicity of nitric oxide that escapes from incomplete hydroxylamine oxidation to nitrite by HAO under fully oxic conditions [78,79], although it is hypothesized that CytS might have a possible role as a nitric oxide-binding protein instead [80]. The expression products of these genes account for the abilities of all the strains to release nitrous oxide when the cultures are maintained in ammonium minimal media [81]. Because urease-encoding genes are also found in the genome of all *N. oceani* strains, all five strains have the genetic capacity to grow on urea as the sources for assimilation of ammonium and carbon as well as the source of ammonia for catabolism [2,82,83].

Prior analyses of the *N. oceani* C-107 genome [2] reported that this species has only single copies of *amo* and *hao* operons while genomes of betaproteobacterial AOB species such as *Nitrosomonas eutropha* C91 [84], *Nitrosomonas* sp. strain AL212 [85], *Nitrosospira multiformis* [86], and *Nitrosomonas europaea* [87] contain multiple operon copies with nearly identical nucleic acid sequences in their genomes [88]. As with C-107, our data revealed that the genomes of all *N. oceani* strains sequenced in this study and in NS58 [32] also have single copies of *amo* and *hao* gene clusters in their genomes. Thus, the notion that *N. oceani* genomes contain only single copies of *amo* and *hao* gene clusters can be generalized. In addition, all *N. oceani* genomes encode the red copper protein nitrosocyanin [89,90], which might play a role in ammonia catabolism by serving as an electron carrier protein [89] or by affecting electron flow indirectly by interaction with HAO or the implicated cytochrome proteins *c*554 or *c*_M_552 [5]. The nitrosocyanin-encoding gene (*nycA*) has, thus far, been discovered exclusively and in all AOB genomes except for the genome of *Nitrosomonas* sp. strain Is79 [91].

All the *N. oceani* genomes also contain the genes encoding cytochrome P460 (*cytL*), another member of high-spin cytochromes *c* [92]. The gene encoding cytochrome P460 was found to be transcriptionally active when grown in the presence of ammonium [5]. The gene product was shown to oxidize hydroxylamine, although at a much lower rate than HAO [93] with the possible pathway of the end production of nitrite by co-oxidation of nitric oxide, possibly in conjunction with NOC_RS04845 homolog pan1-type MCO [2], which is present in other strains as well. This protein was originally hypothesized to be involved in hydroxylamine detoxification [94]; however, the recently proposed model of hydroxylamine oxidation in ammonia-oxidizing Thaumarchaeota [81] implicates this nitric oxide and hydroxylamine co-oxidation chemistry as equally important for nitric oxide detoxification.

#### 3.3.2. Polysaccharide and Glycosyl Transferases

**Figure 6:**
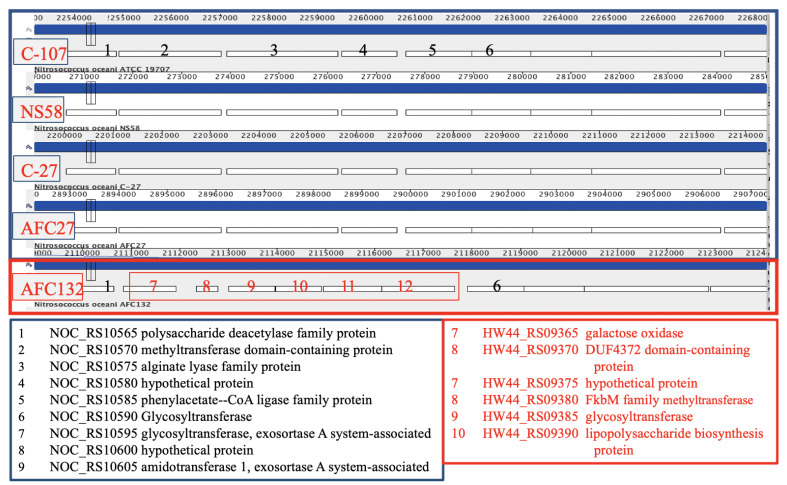
Differences between polysaccharide synthesis and glycosyl transferase encoding clusters at homologous positions in genomes of strains C-107, NS58, C-27, and AFC27 compared with strain AFC132.

All *N. oceani* genomes encode a large complement of glycosyl transferases and inventory involved in polysaccharide biosynthesis; however, there are differences between the strains with regard to extent and location (Figure 6). For example, the gene encoding a capsular polysaccharide (phenylacetate–CoA ligase family protein CDS in the genome of strain C-107 (NOC_RS10585, labeled “5” in Figure 6)) is predicted by the DOOR2 database [95] to be part of an operon containing glycosyl transferase genes. NOC_RS10585 and four preceding genes are replaced in the in AFC132 genome by a longer gene cluster containing genes encoding sulfotransferase, methyltransferase, glycosyl transferase, and inventory implicated in polysaccharide biosynthesis.

The rest of the genes in the operon (starting at NOC_RS10590, labeled “6” in Figure 6) are present in the genomes of all five strains at homologous positions. Gene NOC_RS10440, encoding a polysaccharide biosynthesis protein in the C-107 genome, is missing at the respective position in C-27, NS58, AFC27, and AFC132. In addition, NOC_RS10665 encoding glycosyl transferase found in C-107, NS58, C-27, and AFC27 is substituted by a unique segment in the AFC132 genome containing small hypothetical genes as well as genes predicted to encode glycosyl transferase, glucose-methanol-choline oxidoreductase, and a transposase. Furthermore, a large gene cluster HW44_RS12380-RS12415 containing various glycosyl transferase genes as well as genes encoding polysaccharide biosynthesis and aminotransferase reside in the AFC132 genome in place of a small hypothetical gene in the other strains (NOC_RS13525 in C-107).

The differences in polysaccharide displays between the strains could have a significant impact as a study with *A. macleodii* implicated that complete replacement of gene clusters implicated in synthesis of lipopolysaccharide O-chain, extracellular polysaccharide, and flagellation-related genes in different isolates were reported to create dramatic differences in the cell surfaces of different strains and thus likely provided the ability to produce target-differences for attachment by viruses [59].

### 3.4. Unique Inventory Encoded in the AFC132 Genome

#### 3.4.1. NiFe-Hydrogenase

Based on their main redox metal, hydrogenases are generally categorized in three classes: NiFe-hydrogenase, FeFe-hydrogenase, and Fe-hydrogenases [96]. Of the genome-sequenced *N. oceani* strains presented in this study, only the AFC132 genome was found to encode a putative NiFe-type hydrogenase in Group 3b [96], which is encoded by a unique gene cluster at a locus aligning with the NOC_RS14420 gene on C-107 genome. A sequence homologous to NOC_RS14420 was also detected in the genome sequence of NS58.

A search employing the SyntTax web server [97] identified the presence of NiFe-hydrogenase-encoding genes in the AFC132 genome that were arranged in identical order as found in the genomes of several other bacteria in that the hydrogenase pleiotrophy gene cluster, *hypAFCDE* was succeeded downstream by the structural genes encoding the structural subunits of hydrogenase. The bacteria with homologous NiFe-hydrogenase gene clusters include *Nitrospina gracilis* strain 3/211, a representative of the dominant genus of nitrite-oxidizing bacteria in the marine environment [98] as well as *Marinobacter lipolyticus* SM190, *Marinobacter santoriniensis* NKSG1, *Rhodothermus marinus* SG0.5JP17-172, *R*. *marinus* DSM4252, *Sorangium cellulosum* So0157-2, *S. cellulosum* So-ce56, and *Luminiphilus syltensis* Nor5-1B [99]. Group 3b of the bidirectional (NADP)-dependent hydrogenases [96] of *N. gracilis* is postulated to have the capacity to perform a reversible oxidation of dihydrogen gas using NAD(P)^+^ as the oxidant [100]. This type of hydrogenase is also reported to reduce elemental polysulfide to H_2_S [101]. Although the function of this hydrogenase encoded in the AFC132 genome remains to be tested, there is the possibility that it provides the strain with increased catabolic flexibility by providing the capacity to utilize hydrogen gas as a catabolic source of energy and/or reductant as well as polysulfide reduction as an alternative electron termination process. Interestingly, the genome of the marine nitrifier nitrite-oxidizing bacteria *Nitrococcus mobilis* Nb-231 also contains a gene cluster encoding Group 3b NiFe-type hydrogenase but with a different genetic organization [102].

Other AOB such as *Nitrosococcus halophilus* Nc4 [103], *Nitrosospira multiformis* ATCC 25196 [84], and *Nitrosomonas* sp. Is79 [91] were discovered to have genes required for NiFe-hydrogenase biosynthesis as well. Similar to *N. oceani*, *N. halophilus* Nc4 is also a marine bacterium while the betaproteobacteria *N. multiformis* ATCC 25196 and *Nitrosomonas* sp. Is79 have been isolated from soil and freshwater sediments, respectively. Their putative NiFe-hydrogenases are somewhat different from the one in proposed for AFC132 with the *N. halophilus* Nc4 genome encoding a large hydrogenase subunit containing the F420-reducing domain, while both *N. multiformis* ATCC 25196 and *Nitrosomonas* sp. Is79 genomes encode Group 3d hydrogenases. The diversity in hydrogenases and their overall scarcity in AOB suggest that the NiFe-hydrogenase gene cluster in AFC132 was acquired horizontally at a point in evolutionary time that eludes its determination.

Apparently, not all bacterial genomes with the AFC132-type NiFe-hydrogenase genetic arrangement carry a *hypB* gene. The HypB protein, in concert with HypA, facilitates the maturation process of NiFe-hydrogenase by participation in nickel insertion [104]. This protein has GTPase activity and possesses a CHxnC motif, which binds to either nickel or zinc ([105] and references therein). Of the various species discovered to have the NiFe-hydrogenase genetic arrangement as found in AFC132 in our study, only the two *R. marinus* strains have *hypB* genes that are located at further distance upstream of the NiFe-hydrogenase-encoding gene cluster. OperonDB analysis software [106] predicts that the *hyp*B gene and the gene cluster encoding NiFe-hydrogenase are located in independent operons in *R. marinus* DSM4252. Given the absence of *hyp*B gene in some of these species including *N. oceani* AFC132, it is perhaps that HypB is not critical for the maturation of NiFe-hydrogenase. Several studies have shown that hydrogenase activities of *hyp*B mutants can be partially restored by increased nickel concentration supplementation [107]. Perhaps, nickel insertion into NiFe-hydrogenase can be facilitated by some other enzyme already found in the HypB-negative organism. Homologs of HypB involved in the maturation of other nickel enzymes are known: for instance, UreG participates in the maturation of urease in a similar way as does the accessory protein CooC for the incorporation of nickel into CO dehydrogenase ([108], and references therein). In *Helicobacter pylori*, HypB contributes to nickel incorporation into urease and a *hypB* mutant exhibited lower urease activity due to deficiency of nickel in the urease [105]. Another possibility is that an unknown gene product performs the role of HypB in species lacking the gene encoding typical HypB. Because *N. oceani* AFC132 is urease positive, it is most likely that its UreG protein plays a role in the maturation of the putative NiFe-hydrogenase.

#### 3.4.2. Nonribosomal Peptide Synthetase

All *N. oceani* strains are equipped with the hydroxamate-type siderophore biosynthesis gene cluster NOC_RS09760-RS09775 as reported for the genome of the C-107 type strain [2], which provides the capacity to sequester iron from the environment. However, one of the prominent features with physiological significance and unique to AFC132 is the existence of a putative MbtH-family nonribosomal peptide synthetase (NRPS) gene cluster (HW44_RS01565 (344,153–344,419)) encoding the capacity for siderophore production. The other *N. oceani* genomes only contained pertinent genes required for uptake and processing of the cognate siderophore but not its synthesis. One of these genes, which is conserved at the same locus in all strains, is homologous to TonB-dependent ferrichrome iron receptor, NOC_RS01825. Another gene, NOC_RS01830, is predicted to encode a PepSY-associated trans-membrane helix, which is putatively involved in reduction of ferric siderophores [109]. This could be indication that they have lost the capacity for synthesis and evolved to be cheaters in order to streamline the genome and conserve resources. This trait has been suggested for marine bacteria such as *Vibrio* species, in which selective loss of siderophore biosynthetic genes in conjunction with keeping of the cognate receptor genes seems to have occurred [55]. In addition, this has been suggested for and studied in betaproteobacterial ammonia-oxidizing bacteria from fresh- and wastewater [87,110].

#### 3.4.3. Terpene Synthesis

All *N. oceani* strains share two large gene clusters, NOC_RS05575-RS05740 and NOC_RS07100-RS07285, whose expression products are predicted to be involved in terpene metabolism. Analysis employing antiSmash 2.0 [45] predicted the proteins encoded by these two gene clusters to facilitate ectoine-terpene and phosphonate-terpene synthesis. Another gene, NOC_RS11760, conserved in the C-107, NS58, C-27, and AFC27 genomes appears to be replaced by a unique cluster of genes in the AFC132 genome including gene HW44_RS10660 predicted to encode a geosmin synthase. While a biochemical or physiological function of terpenes in bacteria is elusive, various terpenes are reportedly produced by several marine bacteria [111].

#### 3.4.4. Other uniquely missing or present elements with metabolic implications in the AFC132 genome

Several homologous genes or clusters of genes conserved in the C-107, NS58, C-27, and AFC27 genomes were not identified in the genome of AFC132. For example, the gene NOC_RS07290 encoding peptidoglycan-binding protein LysM that interacts with peptidoglycan hydrolases in bacteria [112] was not identified in the AFC132 genome. Likewise, the gene homologous to NOC_RS00505 and encoding a putative ATPase in the other strains was absent from the AFC132 genome. Remarkably, the cluster of genes NOC_RS02590-RS02610 including a gene encoding glucose-methanol-choline oxidoreductase was absent from the AFC132 genome as well.

Another form of differences between the genomes of AFC132 and the other strains is the replacement of chromosomal sections conserved in C-107, NS58, C-27, and AFC27 with DNA unique to AFC132. One example with putative biological significance is the replacement of a four-gene cluster including NOC_RS06185 predicted to encode DnaJ with a cluster of anti-phage defense ZorAB system (ZorA protein and ompA protein), a hypothetical protein, and a DEAD/DEATH box helicase family protein (HW44_RS05445-RS05460). Likewise, the NOC_RS12755-RS12765 homologous genes conserved in C-107, NS58, C-27, and AFC27 are substituted by two cytochrome *c*-encoding genes in the genome of AFC132. Furthermore, two hypothetical genes NOC_RS08695-RS08700 conserved in C-107, NS58, C-27, and AFC27 are replaced in the AFC132 genome by a gene encoding a putative DOPA 4,5-dioxygenase (HW44_RS07305), an enzyme known to participate in betalain pigment biosynthesis in plants [113]. Despite being identified in some bacterial genomes, the function of this enzyme in bacteria is not yet known [114]. In addition, the region containing the NOC_RS03100-RS03170 homologous gene cluster in the C-107, NS58, C-27, and AFC27 genomes is replaced in the AFC132 genome by a smaller unique segment of several genes including a gene encoding a DNA polymerase III subunit epsilon-like protein and a putative transcriptional regulator.

The AFC132 genome contained unique genes that were absent from the C-107, NS58, C-27, and AFC27 genomes including a gene encoding an additional subunit of RNA-directed DNA polymerase HW44_RS14630.

All *N. oceani* genomes carry a gene cluster encoding a phosphate transport system (NOC_RS12790-RS12825 homologs); however, only the AFC132 genome misses a gene cluster encoding a phosphate transport function. Likewise, only AFC132 does not contain genes encoding homologues to known membrane proteins (NOC_RS03205-RS03225) that could contribute to the formation of a phosphate permease. This finding might mirror the reported genetic difference between *Prochlorococcus* and *Pelagibacter* strains isolated from the P-richer Atlantic and the Pacific Oceans, demonstrating that the genomes of Atlantic isolates were better equipped with phosphate acquisition systems compared to their Pacific relatives [115].

In addition, a gene encoding a putative reverse transcriptase was identified exclusively in the AFC132 genome. The putative gene product encoded in the AFC132 genome is most sequence similar to the retron-type reverse transcriptases belonging to the group II intron family, which are considered diversity-generating retro-elements [116]. This RNA-directed DNA polymerase is known to produce multi-copy single-stranded DNA (msDNA), which is a hybrid DNA-RNA molecule with extensive secondary structures [117]. While msDNA has been hypothetically implicated in epigenetic regulatory events such as social behavior of bacteria, its function in general remains elusive.

### 3.5. Implications of the Genome Differences in the Evolution of Nitrosococcus oceani

There is likely no single one mechanism that determines the holophyletic evolution of bacteria. For example, recombination has been implicated as the main driver of genome divergence in *Vibrio cyclitrophicus* [58] and *A. macleodii* [59], whereas the observed high diversity among *Photobacterium profundum* strains of different bathytypes was attributed, to a large part, to horizontal gene transfers [56]. On the other hand, *C. watsonii* likely diversified by genome degradation, which resulted in the delineation into small-cell and large-cell phenotypes [54].

The high level of content identity and synteny of the *N. oceani* genomes suggests that they have undergone only minimal divergence either after recent distribution to their current geographical locations from the same point of origin or, because this former scenario seems unlikely, the genomes encode effective mechanisms that ensured recalcitrance through evolutionary times. AOB in pure culture never grow beyond threshold densities and their abundance in the natural marine environment is very low [1,3]. Zehr et al. [54,57] suggested that the low genetic diversity observed in bacteria that maintain low abundance such as *C. watsonii* might be a necessary evolutionary strategy adopted by these bacteria to maintain their species identity while more abundant bacteria may derive succession benefits from a higher genomic diversity between the populations in the species [118]. Both scenarios, increasing diversity or high conservation, can be the outcome genomic rearrangement [54] if coupled with pertinent epigenetic mechanisms. Former studies of the catabolic inventory in betaproteobacterial ammonia-oxidizers postulated the operation of rectification mechanisms that ensured near sequence identity between multiple copies of large segments within given AOB cells and a significantly high sequence identity between different populations of strains from different species [4,79,119,120]; however, a closer look into genome divergence between different AOB populations of the same species as reported in this study, has never been attempted. Such rectification mechanisms are likely also at work in the genomes of *Nitrosococcus* genomes, all of which have two copies of rRNA clusters, whereas gene clusters encoding key catabolic functions exist in only one copy [1,2,15,35]. The genomes of *N. oceani* strains C-107, NS58, C-27, and AFC27 as well as AFC132 have an approximate size of 3.5 Mb and a GC content of approximately 50% without displaying a skew for a slightly larger size, a higher number of genes and a lower GC value (C-27 and AFC132) or a smaller size, a lower number of genes and a higher GC value (C-107 and AFC27) that correlates with the location of their isolation (Atlantic: C-107 and C-27; Pacific: AFC27 and AFC132) (Table 1). The draft genomes are larger in size than the closed genome of C-107 except for AFC27, which is potentially due to the applied sequencing technology; however, interestingly, there appears to be a slight skew towards a lower number of CDS in the strains isolated from Pacific Ocean locations with the genome of AFC132 encoding the lowest number of CDS while being the largest genome in size. While the genomes of C-27 and AFC27 differ in size, GC content, and the number of genes, they appear to encode an identical number of CDS, smaller than the number of CDS in C-107, which correlates with the finding that both genomes are high in synteny and identity (Figure 2 and Table 2).

The greater deviation of the AFC132 genome from the other four genomes correlates with a markedly lower number of CDS, the lack of a plasmid and unique deletions and insertions. Many of the locations that indicate deletions and insertions in the AFC132 genome are associated with the residence of transposases, which facilitate genome rearrangements but are also involved in horizontal transfer of free foreign or phage DNA. The plasmids A of C-107, NS58, C-27, and AFC27 contain mostly hypothetical genes, mobile elements, and genes encoding RM systems. Such largely cryptic plasmids may have also played a role in the process of genome evolution, adaptation, and speciation by shuttling genes among the strains [4]. When compared to AFC132, the other *N. oceani* genomes appear to have undergone a process of genome streamlining by getting smaller in size, shedding genes with the capacity to provide greater metabolic capacity (siderophores and NiFe-hydrogenase), and increasing the coding density of the genome (Table 1). Most interesting in this context is that this putative genome economization correlates with a leaner CRISPR/Cas complement when compared with the AFC132 genome. This trajectory of genome economization in marine bacterioplankton was pointed out before by Swan et al. [121].

The phylogenetic tree includes all *N. oceani* strains in one monophyletic clade that shows them most closely related to *N. watsonii* C-113 (Figure 5). In the context of genome content and its economization, it appears most parsimonious that the AFC132 genome closest related to the ancestral lineage of the species and that C-107 represents the most delineated genome with the C-27 and AFC27 genomes in between. Former studies that delegated the evolutionary roots of molecular inventory required for nitrogen-based chemolithotrophy to sulfur-dependent catabolism ([15], and references therein) and the fact that the genus *Nitrosococcus* belongs to the family Chromatiaceae suggests that the genomes of ancestral *Nitrosococcus* species likely encoded a variety of inventory conferring metabolic potential that is now by and large absent from these obligate ammonia-catabolic chemolithotrophs. Hence, the fact that the AFC132 genome still retains the capacity to express NiFe-hydrogenase and an NRPS-encoding gene cluster likely capable of siderophore biosynthesis and that it contains a larger complement of defense inventory lost from the other genomes support the notion of its ancestral status. Genome economization in context with reduced metabolic versatility such as the loss of siderophore production capacity while maintaining the ability for uptake and processing of the cognate siderophore produced other microorganisms (the evolution of cheaters) have been also reported for betaproteobacterial AOB [87]. A search in existing databases did not reveal the origin of plasmid A in the genomes of C-107, NS58, C-27, and AFC27; nevertheless, its stacking with putative toxin–antitoxin systems, a HicB-HicA TA system at NOC_RS00025 and NOC_RS00030, a PemK/MazF TA system at NOC_RS00185, as well as a Type IV TA system at NOC_RS00145 and NOC_RS00175, indicates the stable incorporation of the plasmid in these genomes, which makes it more likely that the plasmid was acquired by the genome ancestral to these three strains than the possibility that the AFC132 genome lost it. Further, the incorporation of the large genomic island in the C-107 and NS58 genomes at a conserved location encoding a transposase in genomes of all other strains suggests that the acquisition of the island occurred after delineation of the C-107 and NS58 genomes from the C-27 and AFC27 genomes.

## 4. Conclusions

The analysis of five genomes representing strains isolated from different oceanic gyres (NS58 and AFC27: from the West and East ends of the North Pacific Gyre, connected clockwise by the Kuroshio, California, and North Equatorial Currents; AFC132: South Pacific Gyre; C-27: coastal waters of the Southern North Atlantic; and C-107: open North Atlantic) revealed extremely low diversity with regard to sequence identity, content, and synteny. Detected differences in genome properties by and large did not disclose a correlation with the location of isolation; nevertheless, strain AFC132 isolated in the South Pacific, which is reported to be less concentrated in oxidized fixed nitrogen, is proposed to be closest related to the ancestor of the species, whereas C-107 and NS58 appear to be the evolutionarily youngest representatives of the species. Given that there are many isolates with near identical sequences of select genes to strain C-107 [1,3], their distribution in the oceans worldwide makes sense in light of the economization of their genomes. In contrast, strains of further delineated species in the genus *Nitrosococcus* have been isolated in distinct locations characterized by high salt concentration (*N. halophilus* [1]) and organics-polluted waters (*N. wardiae* [35]). This distinction also reflects itself in the fact that all strains of *N. oceani* (as well as closely related strains in the species *N. watsonii* but not *N. halophilus* and *N. wardiae*) are ureolytic and urea uptake positive [2,82], which likely reflects an adaptation to the increasingly urea-generating biogenic activities in the Holocene. While it cannot be excluded that one of the strains was transplanted to the different oceanic location, either naturally or inadvertently by human activities, the characteristics illustrated in Table 2 and Figure 2 suggest that the results reflect a general process of genome adaptation in bacteria whose ancestor evolved within a genomic framework that mandates low abundance and is thus basically autochthonous. While this hypothesis has been reported numerous times as cited above, additional research is needed to find causal links between specific genomic inventory and these trajectories of genome evolution.

## Figures and Tables

**Table 1:** General genome characteristics of *Nitrosococcus oceani* strains.

Strains	Chromosomal Genome Size (bp)	Chr. GC%	No. of Plasmid	rRNA	tRNA	^1^ CDS Total	^2^ CDS Orthologous Clusters	^2^ CDS Singletons
C-107	3,481,691	50.32	1	6	46	3641	3581	25
NS58	3,467,956 ^A^ 3,491,291 ^B^	50.33	1	6	45	3641^A^ 3648^B^	3566 ^A^ 3543 ^B^	45 ^A^ 73 ^B^
AFC27	3,471,807	50.30	1	5	44	3610	3508	72
C-27	3,539,918	50.00	1	6	47	3981	3695	281
AFC132	3,545,101	49.80	None	3	46	3956	3227	713

*^1^ RASTtk* [42] and ^2^ *OrthoVenn2* [43]. ^A^ Published data by Dohra et al. [32]; ^B^ re-alignment of the 53 contigs from [32] as described in the methods section.

**Table 2:** Two-way average nucleotide identity (ANI) analysis of *Nitrosococcus oceani* genomes.

Strains	C-107	NS58	C-27	AFC27	AFC132
C-107	-	99.99%	99.99%	99.98%	98.56%
NS58	99.99%	-	99.99%	99.99%	98.28%
C-27	99.99%	99.99%	-	99.99%	98.56%
AFC27	99.98%	99.99%	99.99%	-	98.58%
AFC132	98.56%	98.28%	98.56%	98.58%	-

**Table 3:** Comparison of the CRISPR elements in *Nitrosococcus oceani* strains.

Strains	CRISPR Length (bp)	Direct Repeat (bp)	Direct Repeat Consensus Sequence	Number of Spacers
C-107, NS58, C-27, AFC27	387	28	GTTCACCGCCGCACAGGCGGTTTAGAAA	6
AFC132	2248	28	GTTCACTGCCGCACAGGCAGCTTAGAAA	37

Letters in bold denote difference in nucleotide base.
